# Microparticles Made with Silk Proteins for Melanoma Adjuvant Therapy

**DOI:** 10.3390/gels10080485

**Published:** 2024-07-23

**Authors:** Sonia Trombino, Roberta Sole, Federica Curcio, Rocco Malivindi, Daniele Caracciolo, Silvia Mellace, Dino Montagner, Roberta Cassano

**Affiliations:** 1 Department of Pharmacy, Health and Nutritional Sciences, University of Calabria, Arcavacata di Rende, 87036 Cosenza, Italy; sonia.trombino@unical.it (S.T.); roberta.sole@unical.it (R.S.); rocco.malivindi@unical.it (R.M.); silvia.mellace@gmail.com (S.M.); 2 Department of Experimental and Clinical Medicine, Magna Graecia University, 88100 Catanzaro, Italy; d.caracciolo@unicz.it; 3 Medisilk Spa, San Donà di Piave, 30027 Venezia, Italy; dino@dermasilk.eu

**Keywords:** melanoma, microparticles, silk fibroin, epifibroin 0039, idebenone

## Abstract

Melanoma is one of the most aggressive forms of skin cancer, which is characterized by metastasis and poor prognosis due to the limited effectiveness of current therapies and the toxicity of conventional drugs. For this reason and in recent years, one of the most promising strategies in the treatment of this form of cancer is the use of drug delivery systems as carriers capable of conveying the therapeutic agent into the tumor microenvironment, thus preventing its degradation and improving its safety and effectiveness profiles. In the present work, microparticles based on silk fibroin and epifibroin 0039, silk-derived proteins loaded with idebenone, were created, which act as therapeutic carriers for topical use in the treatment of melanoma. The resulting particles have a spherical shape, good loading efficiency, and release capacity of idebenone. Efficacy studies have demonstrated a reduction in the proliferation of COLO-38, melanoma tumor cells, while safety tests have demonstrated that the microparticles are not cytotoxic and do not possess prosensitizing activity. Notably, transdermal release studies revealed that all particles released idebenone over more days. The analysis of the stimulatory markers of the proinflammatory process, CD54 and CD86, did not show any increase in expression, thus confirming the absence of potential prosesensitization effects of the silk fibroin-based particles. The research, therefore, found that idebenone-loaded silk protein microparticles could effectively reduce the proliferation of melanoma cells without cytotoxicity. This indicates the promise of a safe and effective treatment of melanoma.

## 1. Introduction

Melanoma is a malignant tumor that originates from melanocytes, which are cells that produce the pigment melanin and are present mainly in the skin, but which can also be present in the eyes, ears, gastrointestinal tract, and oral and genital cavities [1,2,3,4,5,6]. Ultraviolet radiation (UVR) is the main, but not the only risk factor, for the onset of melanoma [7]. Other triggering factors that cause this tumor pathology are immunosuppression [8], the presence of nevi, genetic predisposition [9], and obesity [10]. Therapeutic treatment includes surgical resection, chemotherapy, photodynamic therapy, immunotherapy, biochemotherapy, and targeted therapy [11].

In recent years, one of the most promising strategies in the treatment of this tumor form is represented using drug delivery systems as vehicles capable of providing a therapeutic agent to the cancer microenvironment, thus avoiding its degradation and improving its safety and effectiveness. Previous studies have demonstrated the potential of silk fibroin for drug delivery due to its biocompatibility and biodegradability [12,13,14]. For this reason, the application of idebenone-loaded silk microparticles specifically for melanoma therapy has not been fully investigated. In the present work, microparticles based on silk fibroin and epifibroin 0039 (EPI), proteins derived from silk—loaded with the antioxidant idebenone—as therapeutic carriers for cutaneous use for the treatment of melanoma were created.

**Figure 1:**
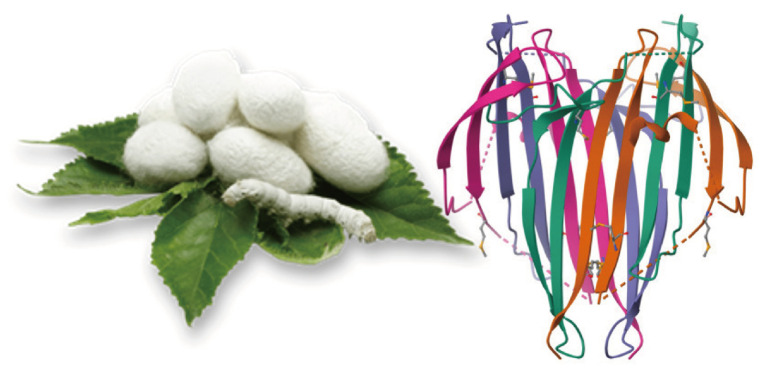
Silk fibroin.

Fibroin (FB) is a protein extracted from the silkworm *Bombyx Mori*. It is mainly composed of the amino acids glycine (43% Gly), alanine (30% Ala), and serine (12% Ser) present in its two subunits: a heavy chain (PM 390 kDa) and a light chain (PM about 26 kDa) connected by a single disulfide bond (Figure 1).

It exhibits very attractive characteristics such as biocompatibility, biodegradability, noncytotoxicity, nonimmunogenicity, ease of surface modification, and stability, thus making it suitable for use as a biomaterial for the formulation of sustained, controlled, pH-dependent, and site-specific drug delivery systems [15,16,17]. Silk fibroin can inhibit the synthesis of melanin, whose excessive production represents a big problem, because theaccumulation of melanin in the epidermis causes serious dermatological problems, such as hyperpigmentation, melasma, freckles, senile freckles, and cancers such as melanoma.

Epifibroin 0039 (EPI) is a medical device, patented by ALPRETEC SRL in Europe, the United States, and Japan, based on 100% pure silk fibroin with permanent antimicrobial protection, thus making it useful for the treatment of skin lesions [18,19].

**Figure 2:**
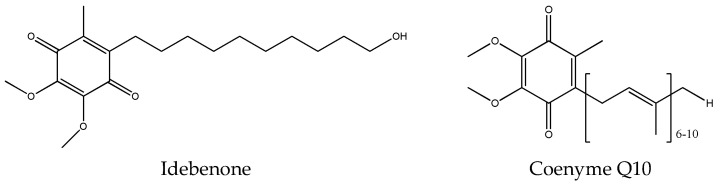
Chemical structure of idebenone (6-(10-hydroxydecyl)-2,3-dimethoxy-5-methyl-1,4-benzoquinone) and coenyme Q10.

The drug loaded in the microparticles based on FB and EPI is idebenone (IDB) (Figure 2). Being one of the most potent antioxidants available for pharmaceutical and cosmetic use, it is a synthetic analog of coenzyme Q10 (Figure 1) capable of suppressing melanin biosynthesis, whose increase causes pathologies such as cancer, as well as glycation and protecting the skin from UV-induced damage [20]. Unfortunately, the unfavorable physicochemical properties of IDB, such as poor water solubility and high lipophilicity, limit its bioavailability after topical administration [21].

Therefore, FB- and EPI-based microparticles could represent a favorable strategy for IBD delivery in melanoma treatment.

The resulting microparticles were characterized for their shape, size, loading efficiency, and idebenone release ability. Efficacy studies have been conducted using COLO-38 human melanoma cells to test the safety and antioxidant and anti-inflammatory activity of these carriers.

## 2. Results and Discussion

### 2.1. Preparation and Characterization of Microparticles

**Figure 3:**
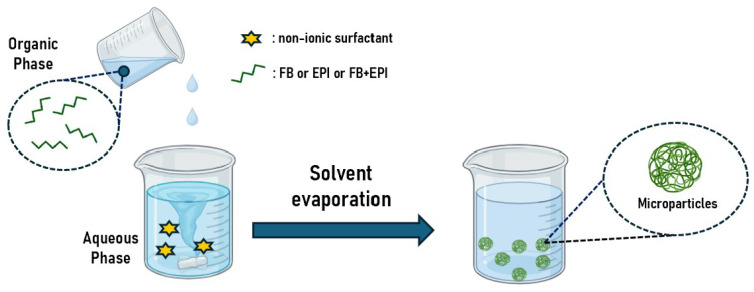
Microparticles production.

The microparticles were prepared using emulsification and precipitation techniques [22], which involve dissolving the polymer in a solvent miscible in water (Figure 3). Then, the polymeric solution was gradually added to another aqueous phase under agitation. Due to the rapid spontaneous diffusion of the polymeric solution into the aqueous phase, microparticles form instantly to avoid water molecules. As the solvent diffuses from the microdroplets, the polymer precipitates in the form of microparticles [23].

The obtained results from dynamic light scattering (Table 1) determined the microparticles’ average diameter, which ranged from 2000 to 3000 nm, and polydispersion index (PI) values indicating good homogeneity in particle size distribution. The implementation of microparticle-sized deliveries was chosen to verify the efficacy of these systems for the treatment of skin tumors, as has already been done in the study by Liu et al. [24].

**Figure 4:**
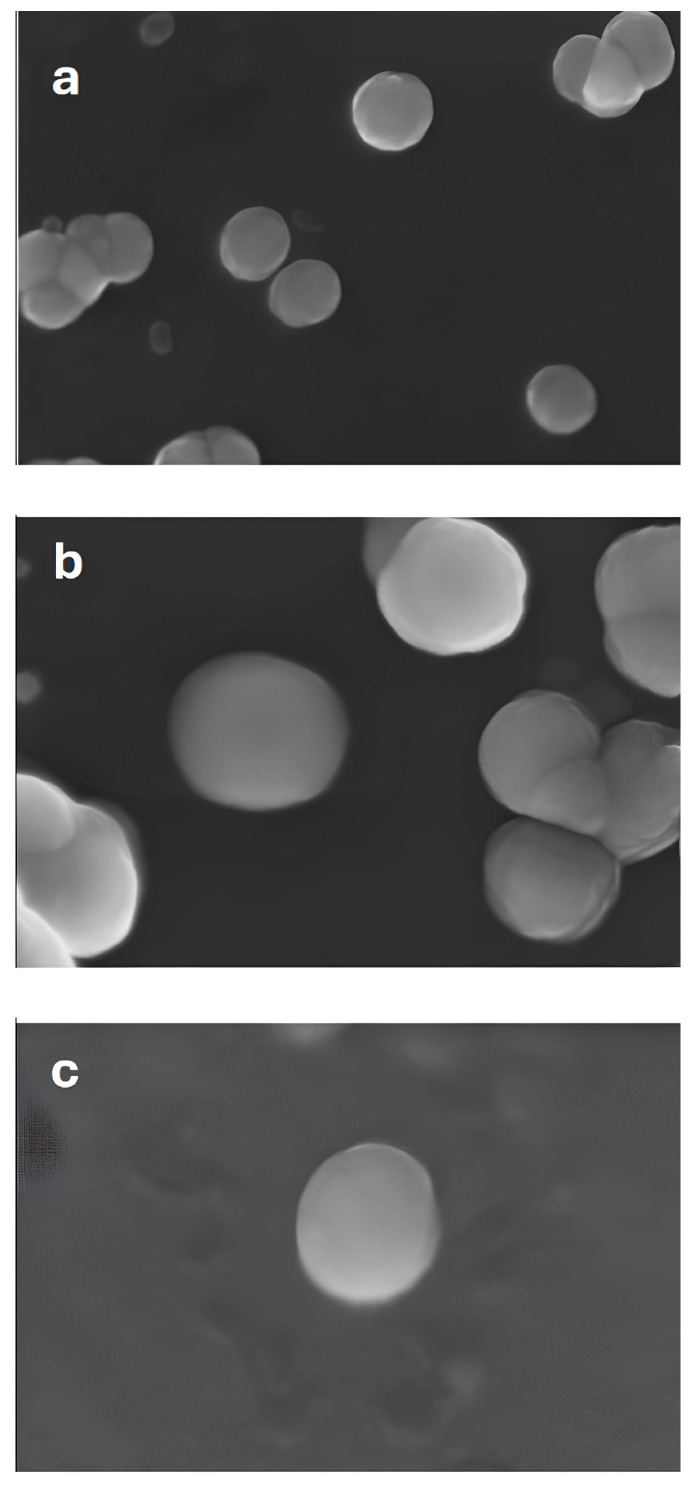
The images were obtained using scanning electron microscopy of (**a**) epi + fibro, (**b**) silk fibroin, (**c**) epifibroin 0039 microparticles.

The images presented in Figure 4, obtained after observation by an optical microscope, show spherical microparticles in the presence of their aggregates. The materials were also observed after six months, and the changes were negligible with respect to measured parameters in terms of shape and size, thus indicating good stability.

### 2.2. Evaluation of Loaded Efficiency

From the microparticles of silk fibroin, epifibroin 0039, and silk fibroin and epifibroin 0039 (both the precipitate and supernatant were examined), the loading efficiency was evaluated through a calculation based on the absorbance data recorded for each formulation. The obtained results are as follows: 58.99% for silk fibroin, 60.81% for epifibroin 0039, 64.60% for the silk fibroin and epifibroin 0039 precipitate and supernatant, and 62.43% for the silk fibroin and epifibroin 0039 precipitate. Each formulation being greater than 50% can be considered as an optimal loading efficiency.

### 2.3. In Vitro Skin Permeation Studies

**Figure 5:**
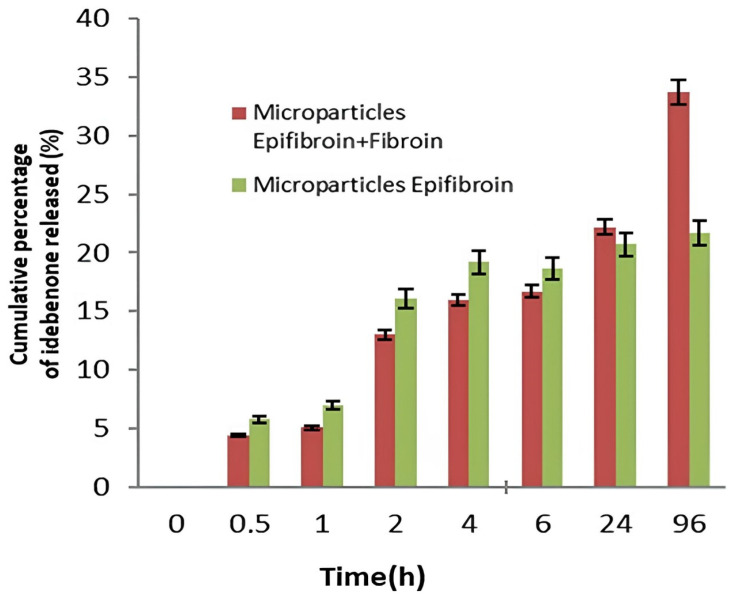
In vitro release profile of idebenone at pH 6.7 and 37 °C. Data are expressed as the mean ± SD of at least three independent experiments.

The release of IDB using silk fibroin and epifibroin 0039 and epifibroin 0039-based microparticles was evaluated at a pH of 6.7 to reproduce the conditions of the tumor environment typical of melanoma. Figure 3 shows the IDB was released in 96 h, more so by silk fibroin and epifibroin 0039 microparticles with respect to epifibroin 0039 ones (Figure 5). This behavior could be ascribed to the establishment of electrostatic interactions within the matrix of microparticles. In the case of epifibroin-based microparticles, these interactions were stronger compared to those established among particles containing both polymers.

### 2.4. Efficacy Testing

Among the several studies that have tested the efficacy of fibroin in antitumoral drug delivery systems, some of them report its activity against melanoma cells [25,26]. In the study by Wang et al. [27], it was demonstrated that silk fibroin has anti-inflammatory activity and the ability to inhibit melanin synthesis in melanoma cells. In Wang’s work, the tests were conducted on B16 cell lines. In relation to this, in our study, the inhibitory activity of fibroin and epifibroin microparticles on melanoma cells other than B16, specifically COLO-38 cells, was investigated. Additionally, it was observed that loading these microparticles with a potent antioxidant such us idebenone amplified the inhibitory activity, thus making it more effective. The mechanism of action of idebenone has not been elucidated, but its role can be better clarified by introducing other antioxidants in the formulation, as well as anti-inflammatory agents without redox activity. In the future, our research may continue to deepen the mechanism of action (redox and consequently anti-inflammatory or only anti-inflammatory) of idebenone.

**Figure 6:**
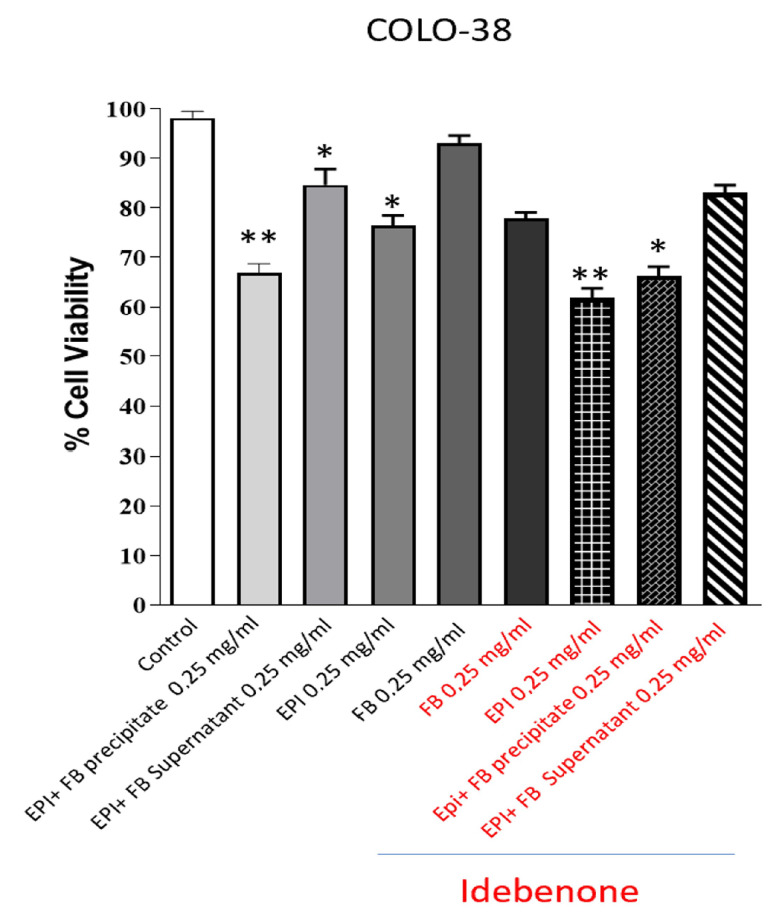
Figure shows the MTT assay results. MTT proliferation assay conducted on COLO-38 cells treated at a dose of 0.25 mg/mL of the tested compounds for 24 h. Results are expressed as a percentage of the average absorbance values relative to the control and represent the mean ± SE of three different experiments. Error bars represent the standard deviation of three independent experiments. Asterisks indicate statistically significant differences compared to the control (* *p* < 0.01, ** *p* < 0.001).

Following the chemical characterization studies of fibroin, epifibroin 0039, and the supernatant and precipitate of fibroin and epifibroin 0039, we investigated whether these compounds could influence the cellular proliferation of our experimental model, COLO-38 cells, using the anchorage-dependent MTT assay. Our results showed that treatment with fibroin, epifibroin 0039, and the supernatant and precipitate of fibroin and epifibroin 0039 (0.5 mg/mL) reduced the incorporation of the MTT substrate. These data demonstrate that the samples in combination, epifibroin and silk fibroin microparticles, can reduce the proliferation of COLO-38 melanoma tumor cells more effectively compared to membranes composed of the same percentages of silk fibroin and epifibroin 0039 (Figure 6).

### 2.5. Safety Testing

#### Evaluation of Cytotoxicity: Neutral Red Uptake (NRU)

**Figure 7:**
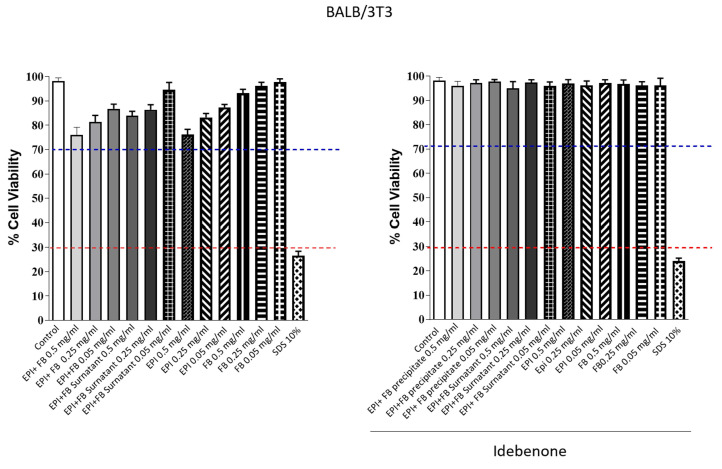
NRU test results. Balb/3T3 Clone A31 fibroblast cell viability (%) measured through NRU cytotoxicity assay upon treatment with increased concentration of different microparticles. Each column represents the means ± SD of 3 wells/group. Red line: strongly cytotoxic, cellular viability < 30%. Blue line: not cytotoxic, 100 < Cellular viability < 70%.

After conducting efficacy studies on silk fibroin, epifibroin 0039, and the precipitate and supernatant of fibroin and epifibroin 0039, safety studies were conducted to assess any cytotoxic and/or prosensitizing effects of our compounds. Regarding cytotoxicity, two types of assessments were employed: one quantitative and one qualitative. In this context, BALB/3T3 cells were treated with microparticles. For the quantitative evaluation using the neutral red uptake (NRU) assay, it was assessed whether the tested compounds could induce cytotoxic effects by measuring the damage caused by the substance to the cellular membranes of fibroblasts. Cell cultures were exposed to different concentrations of fibroin, epifibroin 0039, and the precipitate and supernatant of silk fibroin and epifibroin 0039 (0.5; 0,25; and 0.05 mg/mL) and treated with the neutral red dye, which was retained only by viable and intact cells. Cell viability was subsequently detected through optical density at 540 nm using a microplate reader (Bio-Tek, Winooski, VT, USA). If there was a reduction in cell viability greater than 30% (Figure 6: blue line), it implied that the tested product was cytotoxic. The assay was considered valid if a reduction in viability equivalent to 70% of the positive control (SD 10%; red line in Figure 6) was observed. Figure 7 presents the obtained values, including the positive control integration.

Upon observing the obtained data, it can be established that various microparticles, both empty and loaded with idebenone at the analyzed concentrations, do not exhibit cytotoxic effects. The qualitative assessment was conducted by observing the state of cellular integrity. Biological reactivity (which includes cellular degeneration and potential malformations) was evaluated after 24 h of incubation on a scale from 0 to 4, following ISO 10993-5 standards [28], as shown in the following Table 2.

In Table 3, the values of biological reactivity are reported. Since achieving a numerical grade higher than 2 is considered a cytotoxic effect, it is evident that this assessment has confirmed the nontoxicity of the compounds under investigation.

Regarding the in vitro analysis of the potential prosensitizing effect, the experimentation proceeded with the use of THP-1 monocytes to evaluate the modulation of the expression of two costimulatory molecules involved in the proinflammatory process, namely CD54 and CD86, using nickel sulfate as a positive control, which is a typical contact sensitizing substance. Nickel allows for the elicitation of in vivo immune reactions of an allergenic type, thus making it capable of causing contact sensitization. Therefore, it has been widely used in vitro to study the modulation of the immune response. The recognition of the antigen by the T Cell Receptor (TCR) of T lymphocytes (Signal 1) is not functional to the maturation of an efficient immune response, unless it occurs at the membrane level of an antigen-presenting cell. Both CD54 and CD86 (collectively: B7) are membrane glycoproteins present on the surface of numerous antigen-presenting cells (dendritic cells, Langerhans cells, monocytes/macrophages, and various cell lines—including keratinocytes), acting as costimuli. Indeed, both molecules are ligands of a glycoprotein called CD28, which is present on the membrane of T lymphocytes. The initiation of the ligand/receptor system prevents the apoptosis (programmed cell death) of T cells and cooperates in supporting their proliferation and differentiation. An increase in the expression of these costimulatory molecules on monocytes is a sign of the activation of an immune response following exposure to a potentially sensitizing antigen.

Table 4 reports the results of the analysis of the THP-1 monocyte cell line for the expression of costimulatory molecules using flow cytometry after 24 h of reaction with the sample at a concentration of 0.25 mg/mL and with controls, which were corrected for the negative control. At the concentration examined, the analyzed samples did not show any modulation of the investigated markers. Moreover, they did not exhibit cytotoxic effects on the cells used for the assay, and at the dilutions used, no apoptotic effects were observed on THP-1 cells. Based on the obtained results, the products do not increase the in vitro expression of any of the investigated markers in human monocytes, thus showing no potential for stimulating the immune system mediated by monocyte/macrophages.

## 3. Conclusions

This study aimed to design, produce, and evaluate microparticles based on silk fibroin and epifibroin 0039—both individually and combined. These carriers show potential as transdermal delivery systems for idebenone, a powerful antioxidant, thus making them potentially useful for melanoma treatment. The microparticles showed a good impregnation value of approximately 65%, stability, and dimensions suitable for topical administration, with a diameter between 2000 nm and 3000 nm. Transdermal release studies revealed that all microparticles released idebenone for 4 days. In particular, the carriers formed by epifibroin 0039 and silk fibroin showed a greater capacity for prolonged release. Their potential use as therapeutic carriers was also evaluated through efficacy and safety studies. The MTT test highlighted a reduction in the proliferation of COLO-38 melanoma tumor cells using silk fibroin and epifibroin0039 + IDB microparticles. Safety testing revealed that both empty and drug-loaded carriers are not cytotoxic. Finally, the analysis of the stimulatory markers of the proinflammatory process, CD54 and CD86, did not show any increase in expression, thus confirming the absence of potential prosesensitization effects. The results obtained confirm that idebenone-loaded silk protein microparticles can effectively reduce the proliferation of melanoma cells without cytotoxicity, thus proving to be promising carriers for the safe and effective treatment of melanoma.

## 4. Materials and Methods

### 4.1. Materials

The following substances were used for the synthesis reactions:

Silk fibroin and epifibroin 0039, offered by ALPRETEC SRL Polyvinyl alcohol (PVA) and purchased from Fluka; idebenone (IDE) and PBS (phosphate-buffered saline tablets); acetic acid (CH_3_COOH); ethyl acetate (C_4_H_8_O_2_) and ethanol (C_2_H_6_O) purchased from Sigma-Aldrich (Saint Louis, MO, USA).

The following substances were used for in vitro studies:

COLO-38 human melanoma, BALB/3T3 and THP-1 cell lines, RPMI 1640 (Life Technologies, Inchinnan, UK), trypsin (Sigma-Aldrich, Milan, Italy), phosphate-buffered saline (PBS) (Life Technologies, Monza, Italy) penicillin/streptomycin (Sigma-Aldrich, Milan, Italy), acrylamide and bis-acrylamide (Sigma-Aldrich, Milan, Italy), anti-β-actin antibody (Santa Cruz Biothecnology, Dallas, TX, USA), fetal bovine serum (FBS) (Life Technologies, Monza, Italy), L-glutamine (Life Technologies, Monza, Italy), milk powder (Euroclone), DMEM high glucose (Sigma-Aldrich, St. Louis, MO, USA), HEPES (Sigma-Aldrich, St. Louis, MO, USA), BCS (Sigma-Aldrich, St. Louis, MO, USA), sodium pyruvate (Gibco, UK), β-mercaptoethanol (Sigma-Aldrich, Milan, Italy), MTT (Sigma-Aldrich, St. Louis, MO, USA), DMSO (Sigma-Aldrich, St. Louis, MO, USA), SDS (Sigma-Aldrich, St. Louis, neutral red (Sigma-Aldrich, Milano, Italy), NiSO4 (Sigma-Aldrich, Milano, Italy), CD54 (Invitrogen, Waltham, MA, USA), CD86 (Life Technologies, Inchinnan, UK), FACS buffer (Invitrogen, Waltham, MA, USA), propidium iodide (PI) (Sigma Aldrich, St. Louis, MO, USA).

### 4.2. Instrumentations

UV–Vis spectra were recorded using a Jasco V-530 UV/Vis spectrophotometer using 1 cm thick quartz cells. Scanning electron microscopy (SEM) was carried out using SEM ZEISS Crossbeam 350. Some compounds were freeze-dried through a “Freezing drying” process using a Micro Modulyo from Edwards. A sonicator was used to increase the ethyl acetate solubilization of the samples; centrifugation was carried out by using Thermo Electron Corporation’s “ALC multispeed centrifuge PK21,” which, by causing sedimentation of the microparticles, allows for good separation between precipitate and supernatant. Size analyses of the microparticles were carried out using a Brookhaven 90 Plus Particle Size Analyzer with light scattering (Holtsville, NY, USA).

### 4.3. Preparation of Microparticles

We prepared the microparticles based FB, EPI, and FB + EPI using an emulsion method [29,30,31]. The organic and aqueous phases were prepared separately using an amount of reagents, as reported in Table 5. First, the organic phase was prepared by dissolving FB or EPI or FB+EPI in 10 mL ethyl acetate or acetic acid. An amount of 10 mL of aqueous phase was prepared by dissolving the nonionic surfactant PVA at a concentration of 2%. The organic phase was added to the aqueous phase while stirring at 550 rpm using a magnetic stirrer. The resulting o/w emulsion was sonicated on an ice bath for 1 min (four times at 10 s intervals). The obtained microparticles were stirred continuously for 24 h with a magnetic stirrer, and the organic phase was evaporated through reduced pressure distillation to preserve protein. Then, microparticles were precipitated by centrifugation at 10000 rpm for 45 min, washed, frozen at −80 °C, and lyophilized [31].

### 4.4. Preparation Microparticles Loaded with Idebenone

The microparticles were loaded with IDB using the impregnation technique, which involves the use of a solvent that swells the polymeric matrix and serves as a carrier for the drug [22,32]. In this case, 5 mg of IDB was dissolved in 3 mL of ethanol. The solution was placed inside four flasks, with each containing 10 mg of different polymeric matrix. The sample was subjected to agitation at room temperature and protected from light. After three days, the amount of impregnated IDB was assessed by recovering the drug through centrifugation at 9500 rpm for 10 min; the supernatant was removed and analyzed using UV spectrophotometry to evaluate the drug loading. The impregnated particles were then subjected to drying.

### 4.5. Evaluation of Loading Efficiency

To determine the drug loading efficiency, the supernatant recovered from the impregnation process was subjected to UV–Vis spectrophotometric analysis [22,32]. The measured absorbance values corresponded to the amount of drug not bound to the polymeric matrix. These values were then substituted into the calibration equation for IDB and used to calculate the equation of Loading Efficiency (*%LE*):

%*LE* = (*IDBl* − *IDBf*)/*IDBl* × 100

where *IDBl* represents the drug concentration in the solution before loading, and *IDBf* represents the drug concentration in the solution after its loading.

### 4.6. In Vitro Skin Permeation Studies

Skin permeation tests were performed using Franz diffusion cells (n = 3) with rabbit ear skin (New Zealand rabbits: 2.9–3.1 kg provided by a local butcher) for about 96 h. Franz diffusion cells with a diffusion surface area of approximately 0.85 cm^2^ were assembled, thus maintaining a temperature range between 33.5 and 36.9 °C to replicate physiological conditions. The receptor medium (8 mL) was filled with a PBS solution (pH 6.7.) and ethanol in a 7:3 ratios to enhance IDB solubility, with magnetic stirring to simulate skin conditions. After placing the membranes between the donor and receptor compartments, they were loaded with 0.01 g of different microparticles based on silk fibroin and epifibroin 0039 containing the drug. The solution was sampled from each receptor compartment at regular time intervals (30 min, 1 h, 2 h, 4 h, 6 h, 24 h, 96 h). The samples (8 mL) were collected from the receptor compartment and replaced with fresh receptor medium. The samples were analyzed using UV–Vis spectrophotometry.

### 4.7. Characterization of Microparticles

The microparticles were analyzed using dynamic light Scattering (DLS) and electronic scanning microscopy (SEM). The DLS analysis, used to determinate the dimension of microparticles, involved dispersing an aliquot of microparticles in 10 mL of distilled water at room temperature.

### 4.8. Cell Lines and Culture Conditions

In vitro studies were conducted on COLO 38, BALB/3T3, and THP-1 cell lines, which were maintained in culture with RPMI 1640 1× for COLO 38, DMEM high glucose for BALB-3T3, and RPMI 1640 for THP-1. COLO-38 cells, derived from amelanotic melanoma, were cultured in RPMI 1640 1× medium supplemented with 10% fetal bovine serum (FBS), 1% penicillin/streptomycin, 1% L-glutamine, and 25 mM HEPES buffer. BALB/3T3 cells, murine fibroblasts, were cultured in DMEM high glucose 1× medium supplemented with 10% calf serum (BCS), 1% penicillin/streptomycin, and 1% sodium pyruvate. THP-1 cells, a human monocytic cell line isolated from the peripheral blood of a patient with acute leukemia, were cultured in RPMI 1640 medium with 10% fetal bovine serum (FBS), 1% penicillin/streptomycin, and 0.05 mM β-mercaptoethanol. All cell lines were maintained at 37 °C in a humidified 5% CO_2_-modified atmosphere.

### 4.9. MTT Assay

The MTT assay is a cell proliferation, anchorage-dependent assay that allows for the assessment of cell viability in cell cultures treated with compounds—in this case the microparticles. MTT (3-(4,5-dimethylthiazol-2-yl)-2,5-diphenyltetrazolium bromide) is a yellow-colored substance that, after being reduced to Formazan salts within mitochondria, acquires a violet color. Absorbance is then quantified using a spectrophotometer. Reduction to Formazan salts occurs only if the enzyme is active, thus indicating that it is metabolically active, i.e., there are viable cells. Subsequently, 200 μL of MTT solution was added to each well of a 48-well plate, followed by incubation at 37 °C for 2 h in a humidified 5% CO_2_ atmosphere. The crystals formed by this reaction were then solubilized by adding 200 μL of dimethyl sulfoxide (DMSO) to each well. The color change was subsequently assessed by spectrophotometric analysis at a wavelength of 570 nm.

### 4.10. Neutral Red Uptake Assay

The purpose of the neutral red uptake assay is to study biocompatibility, i.e., to assess in vitro the cytotoxicity of a compound. Living cells can incorporate neutral red into their lysosomes, unlike nonviable cells, where the ability to incorporate neutral red decreases. BALB/3T3 cells were seeded in a 96-well plate at a density of 1 × 10^4^ cells/well and cultured for 24 h at 37 °C in a 5% CO_2_-modified atmosphere. The next day, the culture medium was aspirated from each well, and fresh medium containing microparticles at various concentrations (40%, 60%, 80%, 100%), positive controls (SDS), and negative controls was added. Cells were then incubated again at 37 °C in a 5% CO_2_-modified atmosphere. On the third day, the culture medium was aspirated, and a neutral red (NR) solution was added, followed by a 3 h incubation in a 5% CO_2_-modified atmosphere. At the end of the incubation period, the medium containing neutral red was aspirated, and each well was washed with PBS. In each well, a solubilizing solution (EtOH/acetic acid/deionized water) was added, gently agitated for 10 min, and then read on a spectrophotometer at a wavelength of 540 nm. The test was performed in triplicate for both the sample and control. For each treatment, cell viability was calculated as a percentage relative to untreated controls. If cell viability is >70%, the sample is considered noncytotoxic; if it is less than 70%, the sample is considered cytotoxic.

### 4.11. h-CLAT (Human Cell Line Activation Test)

The h-CLAT test aims to determine whether substances or mixtures cause activation of the immune system, thus indicating skin sensitization. The test was performed on THP-1 monocyte cells—assessing the modulation of CD54 and CD86 expression—and two costimulatory molecules—using nickel sulfate (NiSO_4_) as a positive control. The increase in CD54 and CD86 on monocytes is correlated with the activation of an immune response following exposure to a partially allergenic antigen. THP-1 cells were cultured in RPMI 1640 medium with 10% fetal bovine serum (FBS), 1% penicillin/streptomycin, and 0.05% mM β-mercaptoethanol seeded in a 96-well plate at a concentration of 1.5 × 10^5^ cells per well. After 24 h, the cells were centrifuged, and the medium containing samples and controls was added. The next day, the cells were centrifuged and resuspended in FACS buffer, in the presence of propidium iodide (PI), for analysis by flow cytometry. The CV75, the concentration causing 25% mortality, was then calculated for later use in the actual test. Subsequently, the sample was solubilized in phosphate buffer at a concentration of 100 × of the 1.2 × CV75. Three stock solutions were prepared, with serial dilutions of 1:1.2, in phosphate buffer, thus ranging from 0.335 × CV75 to 1.2 × CV75. These were then diluted 50 times in the culture medium and tested on cells directly with an additional 1:2 dilution. Nickel sulfate served as the positive control at a concentration of 100 μg/mL, while the culture medium was used as the negative control. The experiment was repeated on three different days and conducted in three replicates. After exposure, the cells were centrifuged and resuspended in FACS buffer; they were then divided into three aliquots. They were centrifuged and resuspended in blocking solution (FACS buffer containing 0.01% γ-globulin) and subsequently incubated for 15 min at 4 °C. Finally, the cells were labeled with a fluorescein-conjugated antibody targeting CD86, CD54, or IgG1 (used as a control) for 30 min at 4 °C. Washes were performed with FACS buffer, and additional FACS buffer with propidium iodide (PI) was added. Expression levels of CD54, CD86, and cell viability were then evaluated using flow cytometry.

## Figures and Tables

**Table 1:** Characterization of different microparticles.

Formulation	Size (nm)	Polydispersion Index (PI)
FB	2523 ± 241	0.260 ± 0.024
EPI	2034 ± 173	0.173 ± 0.013
FB+EPI supernatant	2503 ± 201	0.258 ± 0.027
FB+EPI precipitate	3032 ± 352	0.304 ± 0.031

**Table 2:** Guide on interpreting the results.

Level	Reactivity	Conditions of All Cultures
0	None	No detectable area around or under the sample
1	Slight	Some malformed or degenerated cells under the sample
2	Bland	Area limited to the area under the sample
3	Moderate	Area under the sample extends up to 1.0 cm
4	Severe	Area extending beyond 1.0 cm outside the sample

**Table 3:** Biological reactivity assessment.

Samples	Biological Reactivity
Control	0
EPI + FB Precipitate	1
EPI + FB Supernatant	1
EPI	1
FB	0
EPI + FB + IDB Precipitate	0
EPI + FB + IDB Supernatant	0
EPI + IDB	0
FB + IDB	0
Control + (SDS 10%)	4

**Table 4:** Results of the analysis conducted on THP-1 monocytes.

Samples	CD54 *	CD86 *
EPI + FB Precipitate	48.49	61.69
EPI + FB Supernatant	49.15	58.65
EPI	41.49	53.12
FB	49.48	51.52
EPI + FB + IDB Precipitate	51.01	69.68
EPI + IDB Supernatant	49.58	59.15
EPI + IDB Precipitate	48.45	58.48
FB + IDB	55.61	69.59
Control	32	51
Control + (NISO_4_)	179	218

* Cutoff CD86 > 150 e CD54 > 200.

**Table 5:** The amount of substrates required for microparticles.

FB (g)	EPI (g)	PVA (g)	Acetic Acid (mL)	Ethyl acetate (mL)	Distilled H_2_O (mL)
0.01	-	0.2	10	-	10
-	0.05	0.2	10	-	10
0.2	0.02	0.2	-	10	10

## Data Availability

The data presented in this study are openly available in article.
